# A Moveable Feast^1^

**DOI:** 10.3201/eid1611.AC1611

**Published:** 2010-11

**Authors:** Polyxeni Potter

**Affiliations:** Author affiliation: Centers for Disease Control and Prevention, Atlanta, Georgia, USA

**Keywords:** Art science connection, emerging infectious diseases, art and medicine, Nicolaes Gillis, still-life painting, Ontbijtjes, breakfast pieces, Still Life on the Table, enteric infections, foodborne diseases, about the cover

**Figure:**
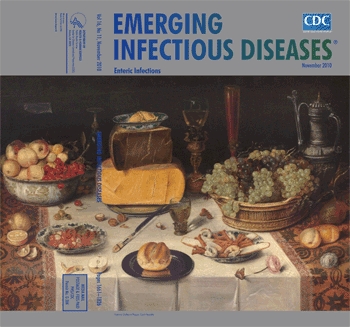
**Nicolaes Gillis (recorded 1601, active 1622–1632 in Haarlem) *Still Life on the Table* (Banquet Table) (1614)** Oil on wood (110 cm × 74 cm) National Gallery in Prague, Czech Republic

A basket of grapes painted by Zeuxis more than 2,000 years ago was so realistic, the grapes so enticing, that birds flew down from the sky to peck at the picture, wrote Pliny the Elder in The Natural History. Food imagery, around since antiquity, expanded and flourished during the 17th century, as part of renewed interest in still-life painting in the Netherlands but also among German, French, Italian, and Spanish artists of the same period.

The rise of still-life painting from the bottom rank of artistic subject matter, especially in the cities of Antwerp, Haarlem, Leiden, and Utrecht, reflected the urbanization of society and a shift in emphasis from religious subjects to domestic scenes featuring personal possessions, earthly pleasures, and the economic prosperity following an explosion of global commerce. Paintings of food became very common, particularly in Haarlem, and formed the basis for later efforts in the genre. *Ontbijtjes*, or breakfast pieces showing foods for a snack or light meal at any time of the day, were wildly popular and witness the eating habits, class distinctions, fads, literary trends, religious beliefs, and health conventions of the period.

The work of Nicolaes Gillis was part of this tradition. Though little is known about his life, Gillis was recorded in Haarlem and painted table or banquet scenes, one of the first in his generation to do so, along with Floris van Dijck and Floris van Schooten. These artists probably knew each other’s work very well. Their mostly two-dimensional compositions showed tables arranged strictly parallel to the horizontal edges of the canvas and were additive in their presentation and arrangement of objects. Gillis may have been the oldest of the group―a 1601 date appears on one of few works by him that have survived.

*Still Life on the Table*, on this month’s cover, is a breakfast piece showing fruits, nuts, bread, and cheeses served as dessert. Like similar paintings in this vein, it offers a glimpse of graceful living. A delicate table runner with crocheted border has been unfolded over the tablecloth for the occasion, the creases carefully running toward the back of the painting, creating the illusion of perspective. A slightly downward view allows good overall display of all the objects on the table, which are arranged side by side, touching only here and there, rarely overlapping, placed all the way to the edges of the canvas, reaching out to the viewer.

Fruit, though not an important part of the local diet, is abundantly displayed because of its visual appeal. This appeal, expertly demonstrated in Caravaggio’s simple basket of fruit back in 1596, never lost its popularity. “Fine and rare fruits of various kinds are usually chosen for the dessert, for the pleasure they afford by their contrasting beauty.” Lavish goblets and serving pieces provide richness and glow around a pyramid of hard cheeses. Floris van Dijck also often stacked these in two or more layers. The bottom comprises half a wheel with deep yellow color, still young. The top is smaller and darker, showing an aged, more mature product. Cheese, a common food eaten in large quantities by the masses, was also enjoyed by the affluent as part of dessert. Later, in his Physiology of Taste (1825), Jean Anthelme Brillat-Savarin would uphold this convention: “A dinner that ends without cheese is like a beautiful woman with one eye.”

While in early banquet paintings the food seemed untouched, Gillis’ work contains signs of consumption, a feeling of spontaneity, and bits of realism. The lemon has been cut, the seeds clearly visible; some of the fruit is peeled, the skin still attached; nut shells are scattered on the table; and along with the flower in the forefront rests a moth. The cheeses have irregular cuts made by the knife now on the platter. While food is beautiful, eating is a human need not usually included in food paintings, except perhaps in extreme situations, as in Hieronymus Bosch’s *Garden of Worldly Delights*, which actually shows a figure, a half-human bird demon, eating in hell. Seventeenth-century Dutch and Flemish paintings also sometimes depicted peasants as stupid and ugly creatures eating and sometimes vomiting.

Banquet tables laden with costly exotic food and gleaming accessories have received many and sundry interpretations, apart from the obvious one of luxury. Some have dwelled on the moral undertones, suggesting the transitory nature of worldly pleasures―an argument against indulging in them―although the beauty and seductiveness of the displays argue in favor of the food. Others have commented on religious symbolism, the Lenten nature of meatless compositions, although fasting and feasting have long been argued among the various religious sects. Yet others remind that in the Golden Age, as in our times, food was often linked to health. Balance in meals was held in high regard, and foods were believed to complement each other. For this reason, game and lobsters were often paired in paintings.

Fascination with the beauty of food and interest in its connection with health remain intense and, in our times, go far beyond pairing food items, although debate on nutrition is increasing along with the growing epidemic of obesity in the United States and abroad. While not as photogenic, the utilitarian act of eating, including food consumed, and on the other end of the alimentary canal, food depleted, is fraught with danger and remains extremely central. Consumers are plagued by illnesses, among them enteric infections. The emphasis over the years has been in the prevention of foodborne disease—from the ubiquitous outbreaks of salmonellosis to more exotic fare due to new and reemerging foodborne pathogens.

But enteric infections, among them cholera and typhoid fever, until the 19th century, generally were not associated with food but rather with a variety of unsavory and unsanitary conditions. These and many other enteric infections, also not always foodborne, are with us today―some, and their complications, in this issue: *Salmonella enterica* clusters in Minnesota, extended spectrum β-lactamase–producing *Escherichia coli* in neonatal care, decreased ciprofloxacin susceptibility in *S. enterica* infections and association with foreign travel, multidrug-resistant *S. enterica* serovar Infantis in Israel, *Shigella* spp. antimicrobial resistance in Papua New Guinea, geographic expansion of *Baylisascaris procyonis* roundworms in Florida, and intentional infection with probiotics that can become invasive. Some enteric infections due to noroviruses, the most common causes of gastroenteritis in the world, also not always foodborne, are at times spread through contaminated food.

“If you are lucky enough to have lived in Paris as a young man,” wrote Ernest Hemingway in his Memoirs, “then wherever you go for the rest of your life, it stays with you, for Paris is a moveable feast.” The same can be said of new and reemerging infections. Prevention and control have curbed and tamed some, but the threat remains. Like a moveable feast, they are present but on their own time. And in different forms and unexpected ways, they always stay with us.
